# In Vitro Biocompatibility of the Novel Ceramic Composite Baghdadite for Defect Augmentation in Revision Total Hip Arthroplasty

**DOI:** 10.3390/jfb14100517

**Published:** 2023-10-15

**Authors:** Max Jaenisch, Christian Guder, Robert Ossendorff, Thomas M. Randau, Sascha Gravius, Dieter C. Wirtz, Andreas C. Strauss, Frank A. Schildberg

**Affiliations:** 1 Department of Orthopedics and Trauma Surgery, University Hospital Bonn, 53127 Bonn, Germany; 2 Department of Orthopedics, Orthopedic Surgery and Sports Medicine, Augustinian Hospital Cologne, 50678 Cologne, Germany; 3 Department of Orthopedics and Trauma Surgery, University Medical Center Mannheim of University Heidelberg, 68167 Mannheim, Germany

**Keywords:** ceramic bone substitute, Baghdadite, cytotoxicity, MG-63, THP-1, inflammation, revision total hip arthroplasty

## Abstract

Biological augmentation of bony defects in weight-bearing areas of both the acetabulum and the femur remains challenging. The calcium-silicate-based ceramic Baghdadite is a very interesting material to be used in the field of revision total hip arthroplasty for the treatment of bony defects in weight-bearing and non-weight-bearing areas alike. The aim of this study was to investigate the biocompatibility of Baghdadite utilizing an osteoblast-like, human osteosarcoma cell line (MG-63) and the human monocytic leukemia-derived cell line (THP-1). THP-1-derived macrophages and MG-63 were indirectly exposed to Baghdadite for 7 days using a transwell system. Viability was assessed with MTT assay and pH analysis. To investigate proliferation rate, both cell lines were labelled using CFSE and flow cytometrically analyzed. ELISA was used to measure the secretion of IL-1ß, IL-6 and TNFα. The investigation of viability, while showing a slight difference in optical density for the MTT assays in MG-63 cells, did not present a meaningful difference between groups for both cell lines. The comparison of pH and the proportion of living cells between groups did not present with a significant difference for both THP-1 and MG-63. Baghdadite did not have a relevant impact on the proliferation rate of the investigated cell lines. Mean fluorescence intensity was calculated between groups with no significant difference. Baghdadite exerted a proinflammatory effect, which could be seen in an upregulated production of TNFα in macrophages. Production of IL-1ß and IL-6 was not statistically significant, but the IL-6 ELISA showed a trend to an upregulated production as well. A similar effect on MG-63 was not observed. No relevant cytotoxicity of Baghdadite ceramics was encountered. Baghdadite ceramics exhibit a proinflammatory potential by significantly increasing the secretion of TNFα in THP-1-derived macrophages. Whether this proinflammatory potential results in a clinically relevant effect on osteointegration is unclear and requires further investigation. Baghdadite ceramics provide an interesting alternative to conventional bone substitutes and should be further investigated in a biomechanical and in vivo setting.

## 1. Introduction

Revision total hip arthroplasty (RTHA) is a challenging field of practice with a projected increase in frequency especially for young patients [1]. Aseptic loosening is the most common cause of failure often resulting in large osseous defects of the surrounding host bone stock [2]. In revision arthroplasty of the hip joint, augmentation of the insufficient bone stock is required to achieve a long-term stable implant fixation [3,4]. While metallic, macro-porous augmentation is gaining popularity, the ultimate goal of RTHA should be biological defect downsizing. To achieve biological defect downsizing, various techniques of autologous and allogenic bone grafts—in bulk or morcalized configurations—have been published [5].

The limitations of autologous and allogenic bone grafts are availability, high cost and osseointegration. For allogenic bone grafts, donor disease transmission, immunological reactions and donor site morbidity are also concerning possibilities for complications. Impaction bone grafting is a well-established technique for the treatment of bone defects in non-weight-bearing areas of the acetabulum and femur. Autologous or allogenic cancellous bone croutons and chips are combined with the blood of the patient and are impacted into the defect. A metallic implant (e.g., reconstruction cage) is implanted on top to protect the bone graft until it is integrated [6]. Depending on defect size and especially in cases of re-revision, large quantities of bone graft are required. Biological augmentation of bony defects in weight-bearing areas of both the acetabulum and the femur remains challenging. While impaction bone grafting cannot be applied in weight-bearing areas, bulk allografts (autologous or allogenic) have been used throughout the years. However, in contrast to promising short term results, multiple analysis of long-term outcome showed a high rate of failure with limited integration, fibrous encapsulation and consecutive resorption and failure [7,8,9,10].

Due to the limitations of autologous and allogenic bone grafts, as mentioned above, the research into bone substitute materials is on-going. The perfect bone substitute for revision arthroplasty would be low-cost, biocompatible, promote osseointegration and be available in multiple configurations (morcalized, scaffold). Porous ceramic scaffolds are considered one of the most critical implants for tissue engineering applications in the field of RTHA [11]. The porous structure facilitates the exchange of oxygen and nutrients, the disposal of waste products as well as osteogenesis and vascular invasion into the pores [12]. In addition, the porous surface could facilitate mechanical interlock between the scaffold and the surrounding host bone similar to trabecular metal augments [13]. To be used as a scaffold in weight-bearing areas, the bone substitute would also need to be biomechanically stable, biodegradable, and form-adaptable to individual defect configurations in a porous structure (scaffold).

To meet these requirements, bioceramics have been extensively researched in the recent past. The material group of bioceramics is defined by their ceramic composition and biocompatibility. From this large group of compounds, calcium phosphates (CaP) and silicate-based ceramics have been extensively studied for their potential to be bioactive. In this context, bioactivity refers to the ability to form hydroxycarbonated apatite when exposed to body fluid, which makes up the majority of human bone tissue. Furthermore, they have been introduced in a wide range of clinical applications, including bone reconstruction, toothpastes, dermal fillers and formulations for soft tissue regeneration [14]. However, these conventional bioceramics have limited applications for the reconstruction of critical-sized bone defects in weight-bearing areas due to their limited mechanical strength and fracture toughness [15]. Recently, the reinforcement of CaP and silicate-based ceramics with different ions or metal oxides has been introduced to improve their mechanical and biological properties. This novel class is called “doped” bioactive ceramics and offers a unique set of properties.

The addition of magnesium to CaP can result in improved Young’s modulus, maximum temperature, and ultimate strength [16]. The reinforcement of CaP by Strontium nitrate can increase grain and particle size [17]. Other doped bioceramics such as Akermanite, Sr-Hardystonite and Sr-HT-Gahnite have shown satisfactory structural, mechanical and biological properties for bone regeneration [18]. Other possible effects through the addition of bioactive ions may be enhanced cell proliferation and expression of genes related to osteogenesis and angiogenesis [19].

Another promising contender is Baghdadite, a calcium-silicate-based ceramic, which incorporates Zirconium (Zr), firstly introduced in 2008 by the group around Hala Zreiqat in Sydney, Australia [20]. Baghdadite has been reported to offer improved physical and mechanical properties when compared to conventional calcium-silicates due to the addition of Zr, which is a quadrivalent ion and can be linked to Ca ions [21]. To enable load transmission to the remaining stable host bone and minimize stress shielding, the elastic modulus of a bone substitute scaffold in weight-bearing areas should be analogous to that of the bone tissue to reduce resorption and degradation [22]. Baghdadite has been reported to meet the lower end of the range for trabecular and cortical bone [12]. Additionally, the ability to manufacture Baghdadite scaffolds through 3D printing has been described and is similar to the production of trabecular metal augments and individualized implants being used for RTHA [23]. Therefore, already existing planning and production pathways could be utilized. Due to these properties, Baghdadite is a very interesting material to be used in the field of RTHA for the treatment of bony defects in weight-bearing and non-weight-bearing areas alike.

The aim of this study is to investigate the biocompatibility of Baghdadite utilizing a human monocytic leukemia-derived cell line (THP-1) and osteoblast-like, human osteosarcoma cells (MG-63). We tested for viability, ability to proliferate and inflammation mediators.

## 2. Materials and Methods

### 2.1. Cell Culture

The human monocytic leukemia-derived cell line THP-1 (American Type Culture Collection, Manassas, VA, USA) and the osteoblast-like, human osteosarcoma cell line MG-63 (American Type Culture Collection, Manassas, VA, USA), were cultivated in RPMI 1640 medium supplemented with 10% fetal bovine serum and 1% penicillin/streptomycin at 37 °C in a humidified atmosphere with 5% CO_2_. To trigger macrophage differentiation, the THP-1 cells were stimulated with 50 ng/mL phorbol 12-myristate 13-acetate (PMA) (Sigma-Aldrich, Taufkirchen, Germany) overnight. Dense Baghdadite microplates with a diameter of 2 mm were added to the corresponding wells using a transwell system, thus allowing the cellular response to be studied in indirect contact without causing any mechanical stress on the cells.

### 2.2. Preparation of Baghdadite

Baghdadite (Ca_3_ZrSi_2_O_9_), a calcium-silicate-based ceramic, was synthesized by sol-gel method using zirconia oxide nitrate (ZrO(NO_3_)_2_), calcium nitrate tetrahydrate (Ca(NO_3_)_2_·4H_2_O) and tetraethyl orthosilicate (TEOS, (C_2_H_5_O)4Si) as raw materials. TEOS, ethanol and HNO_3_ were mixed at a mol ratio of 1:8:0.16 and hydrolyzed under stirring for 30 min. ZrO(NO_3_)_2_ and Ca(NO_3_)_2_·4H_2_O were added at a mol ratio of 1:3:2 (ZrO(NO_3_)_2_/Ca(NO_3_)_2_·4H_2_O/TEOS). The reactants were stirred for 5 h at room temperature. The solution was maintained for 1 day (60 °C) and dried to obtain dry gel for another 2 days at 100 °C. Calcination was carried out at 1150 °C for 3 h. The Baghdadite was manufactured as cell culture microplates with a diameter of 2 mm. To achieve this, the calcinated Baghdadite powders were sieved to 230 meshes and mixed with 6% (*w*/*v*) polyvinyl alcohol water solution binders using a 1:9 weight ratio of PVA solution and Baghdadite powders. To finalize the process, the mixture was pressed uniaxially at 200 MPa into the form of microplates and sintered at 1400 °C for 3 h with a heating rate of 2 °C/min.

### 2.3. MTT Viability Assay

An MTT assay was used to indirectly measure the growth properties of the THP-1 and MG-63 cell lines. The corresponding cells were cultured at a density of 25,000 cells/well for THP-1 cells and 2000 cells/well for MG-63 cells in a 96-well plate as a monolayer culture under standard conditions. The culture medium was changed twice per week. The measurements were carried out at the indicated time points according to the manufacturer’s protocol of the MTT assay kit (Boster Biological Technology Co., Ltd., Pleasanton, CA, USA).

### 2.4. CFSE Proliferation Assay

To assess proliferation rate, THP-1 and MG-63 cells were labeled using carboxyfluorescein succinimidyl ester (CFSE) (Molecular Probes, Leiden, Netherlands) to track CFSE dilution over time and thereby examine cell division. The cells were cultured at a density of 200,000 cells/well for THP-1 cells and 15,000 cells/well for MG-63 cells in a 96-well plate as a monolayer culture under standard conditions in the presence or absence of Baghdadite. Cell proliferation was flow cytometrically assessed by analyzing the CFSE dilution. A BD FACS Canto II cell analyzer and FlowJo software (BD Biosciences, Heidelberg, Germany) were used.

### 2.5. Enzyme-Linked Immunosorbent Assay

PMA-differentiated THP-1 and MG-63 cells were treated with Baghdadite for 1 day, 2 days, 5 days, and 7 days. Cell-free supernatants were collected and centrifuged (200× *g*, 10 min, 4 °C), and aliquots were stored at −80 °C. The production of the cytokines TNFα, IL-1ß and IL-6 was determined with an ELISA kit according to the manufacturer’s protocol (R&D Systems, Wiesbaden, Germany) using a microplate ELISA reader.

### 2.6. pH Analysis

The corresponding cells were cultured at a density of 25,000 cells/well for THP-1 cells and 2000 cells/well for MG-63 cells in a 96-well plate as a monolayer culture under standard conditions. pH values were measured in the cell culture medium at the indicated time points in the presence or absence of Baghdadite.

### 2.7. Statistical Analysis

For the statistical analysis GraphPad Prism 7 software (GraphPad, La Jolla, CA, USA) was used. All values are reported as the mean ± SD. The Shapiro–Wilk test was used to test for normal distribution. For data with normal distribution, Student’s *t*-test was used for comparison between two groups. One-way ANOVA was used to determine statistical difference between several groups. Mann–Whitney U testing was used for not-normally distributed data. The level of significance was set at * *p* < 0.05, ** *p* < 0.01, and *** *p* < 0.001.

## 3. Results

In this study, we evaluated the biocompatibility of the calcium–silicate–zirconium compound Baghdadite. We utilized a human monocytic leukemia-derived cell line (THP-1) and an osteoblast-like, human osteosarcoma cell line (MG-63) to mimic both an immediate immune response, and the reaction of a bone-producing cell line.

### 3.1. Viability

**Figure 1:**
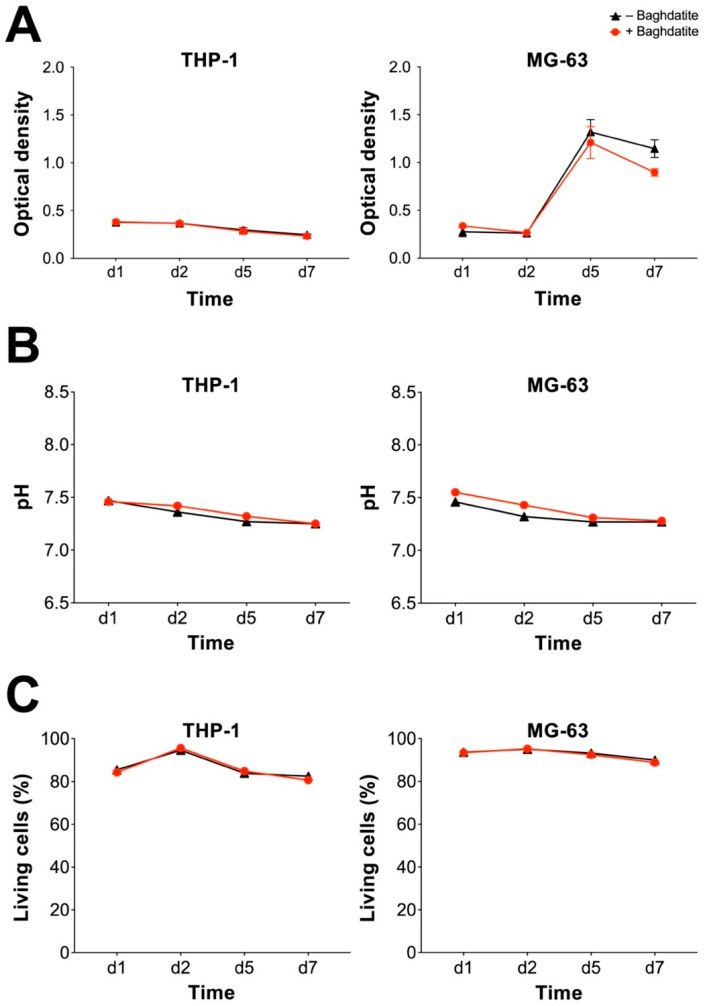
Cytotoxic potential of Baghdadite test bodies on THP-1 and MG-63. OD of MTT assay (**A**), pH of culture medium (**B**) and percentage of live cells (**C**) in the evaluation for THP-1-derived macrophages (**left**) and MG-63 human sarcoma cells (**right**). No significant differences were observed between the presence and absence of Baghdadite.

Analyzing cell viability is crucial to rule out relevant cytotoxicity. Macrophages have a multifaceted role in bone healing and can facilitate inflammation as well as regeneration. A complete failure of bone healing was shown if macrophages were depleted [24]. Therefore, the survival of both macrophages and osteoblasts is essential to enable proper osseointegration of implants and scaffolds. THP-1-derived macrophages and MG-63 human osteosarcoma cells were exposed to a Baghdadite test body for up to 7 days. An MTT assay was carried out and did not display any significant difference in optical density (OD) between groups for THP-1 macrophages (Figure 1A). For both groups, the OD decreased slightly over time. MG-63 cells showed an increase in OD for both groups (Baghdadite and control) due to the strong proliferative behavior of the cell line. No significant difference was found between the Baghdadite and control groups. The comparison of pH and the proportion of living cells between groups did not present with a significant difference for both THP-1 and MG-63 (Figure 1B,C). The comparison of viability did not present a meaningful difference between groups for both cell lines.

### 3.2. Proliferation

**Figure 2:**
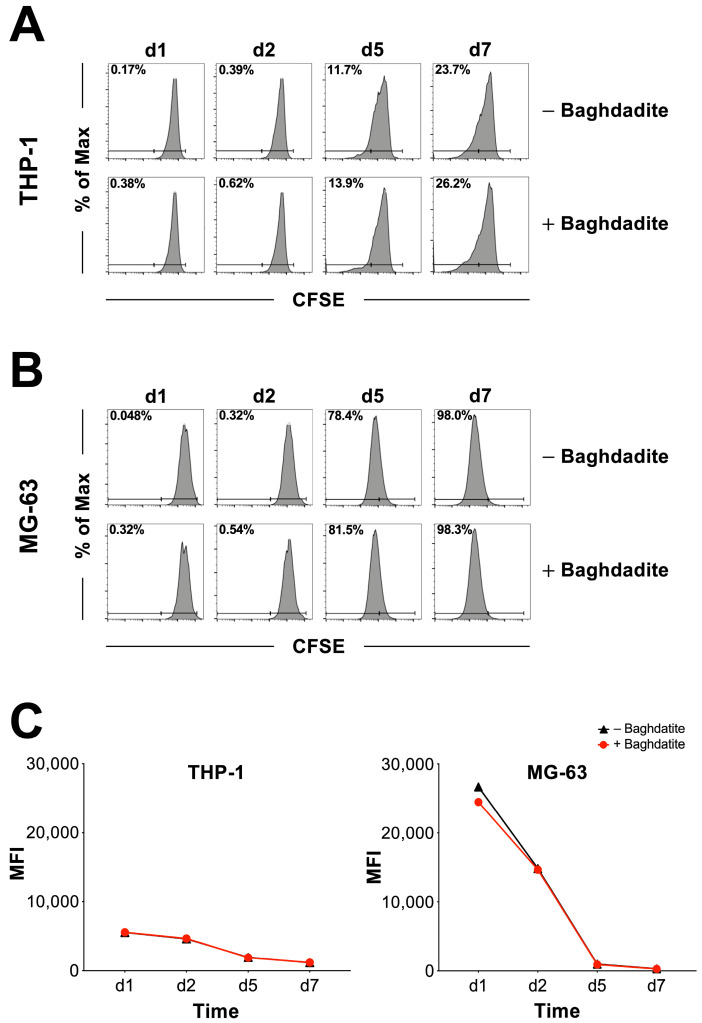
Analysis of cell proliferation rate. Proportion of proliferating and non-proliferating cells based on CFSE proliferation assay for THP-1-derived macrophages (**A**) and MG-63 human sarcoma cells (**B**). Part (**C**) shows the mean fluorescence intensity for THP-1-derived macrophages (**left**) and MG-63 human sarcoma cells (**right**). No significant differences were observed between the presence and absence of Baghdadite.

To evaluate the rate of cell division in the presence and absence of Baghdadite, THP-1 and MG-63 cells were labeled with CFSE and evaluated at different time points. Loss of CFSE intensity was investigated and cells were analyzed using a flow cytometer. Figure 2 shows the percentage distribution of proliferating and non-proliferating cells sorted by groups (Baghdadite −/+) for THP-1-derived macrophages (Figure 2A) and MG-63 human osteosarcoma cells (Figure 2B). Both THP-1-derived macrophages and MG-63 human osteosarcoma cells did not display a meaningful difference in proliferation percentage between groups. The presence of a Baghdadite test body did not appear to have a relevant impact on the proliferation rate of the investigated cell lines. To further quantify any potential difference in proliferation rate between groups, the corresponding mean fluorescence intensity was calculated (Figure 2C) with no significant difference.

### 3.3. Proinflammatory Activity

**Figure 3:**
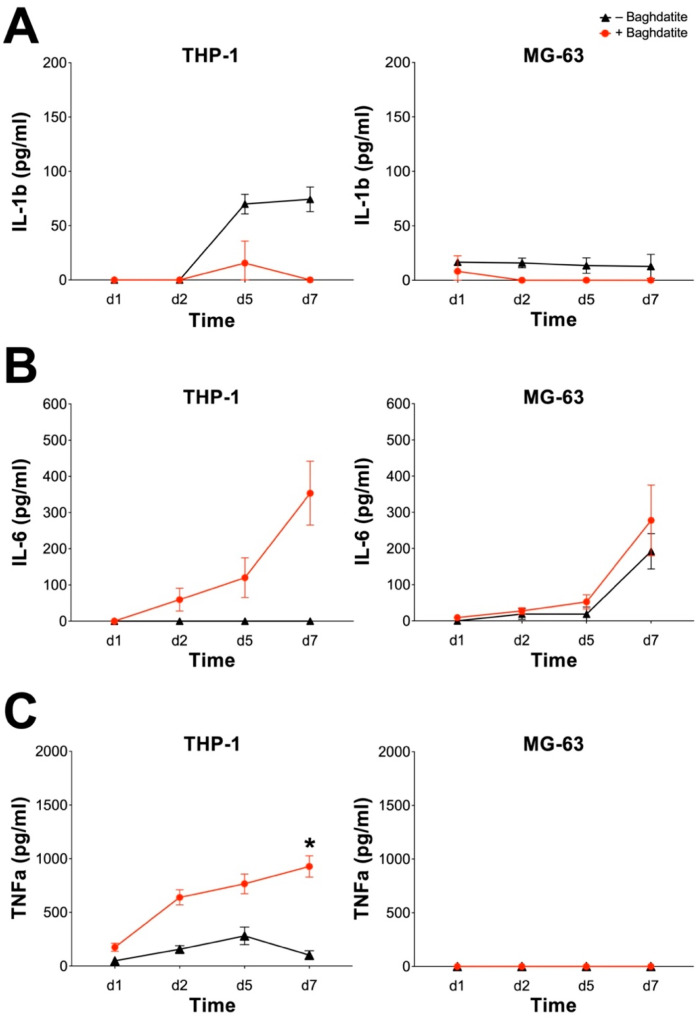
Proinflammatory potential of Baghdadite on THP-1-derived macrophages and MG-63 human sarcoma cells. The THP-1-derived macrophages and MG-63 cells were incubated with Baghdadite test body for up to 7 days. Release of IL-1ß (**A**), IL-6 (**B**) and TNFa (**C**) was measured by ELISA for THP-1-derived macrophages (**left**) and MG-63 human sarcoma cells (**right**). * indicates significant differences between presence and absence of Baghdadite (* *p* <0.05).

A common problem in orthopedics and trauma surgery is aseptic loosening. This process can be caused by a chronic inflammation by inducing osteoclastic bone resorption and suppressing bone formation [25]. This inflammatory reaction can be triggered by various byproducts of joint replacements and is mainly driven by macrophages. Among all proinflammatory mediators, IL-1ß and TNFα are the primary initiators and significant mediators of this inflammatory cascade [26]. IL-6 can be produced by macrophages and osteoblasts alike and has been reported to stimulate osteoclast formation and bone resorption [27]. We, therefore, investigated the proinflammatory effect of Baghdadite by evaluating the induction of IL-1ß, IL-6 and TNFα on the THP-1 and MG-63 cell lines (Figure 3).

IL-1ß secretion was detected in both cell lines with and without a Baghdadite test body (Figure 3A). THP-1-differentiated macrophages secreted slightly increased amounts of IL-1ß starting at day 2, which might be due to the differentiation process. However, this phenotype was not seen in the presence of Baghdadite, and there was no significant difference between its absence or presence. MG-63 cells in both groups exhibited only minimal secretion of IL-1ß. IL-6 production was enhanced in THP-1 macrophages through the addition of Baghdadite with a solid increase after day 5. These results, however, did not prove statistically significant in comparison to the control group (Figure 3B). Although there was a general trend for MG-63 to produce more IL-6 over time in culture in the absence or presence of Baghdadite, no significant differences were observed between the two groups. TNFα production was significantly increased in the presence of Baghdadite in THP-1 macrophages. The increase was steady in the Baghdadite group, with the highest measurement on day 7, while the TNFα secretion of the control group increased only slightly, resulting in a significantly different TNFα level between the control and Baghdadite groups. The MG-63 cell line did not produce measurable TNFα level with or without Baghdadite (Figure 3C).

In summary, the presence of Baghdadite may result in a proinflammatory effect on macrophages resulting in an upregulated production of IL-6 and TNFα. However, only the difference in TNFα production was statistically significant. A similar effect on MG-63 was not observed.

## 4. Discussion

Cases of RTHA often present with large uncontained defects of the weight-bearing area of the acetabulum and of the stability-generating aspects of the femur. Biological augmentation and consecutive defect downsizing have been challenging and often result in suboptimal long-term clinical results. Therefore, the idea of a mechanically stable, 3D-printable, and biocompatible ceramic bone substitute is exciting for surgeons and researchers alike.

However, care must be taken to evaluate each aspect separately before progressing to an in vivo model. In this publication, we investigated the biocompatibility of Baghdadite with the focus on a later employment in the field of RTHA. Our group is made up of surgeons and basic science researchers combining scientific experience with clinical expertise. We present a structured and easily reproducible experimental setup.

Our study reported good biocompatibility of a Baghdadite test body without significant differences in cytotoxicity and proliferation rate between groups after 7 days. Other in vitro experimental setups support these results. The group around Arefpour et al. examined the behavior of Baghdadite nanoparticles on mesenchymal bone marrow stem cells and reported no significant cytotoxicity [28]. Ramaswamy et al. investigated the effect of Baghdadite ceramics on the proliferation and attachment of human osteoblast-like cells (HOB), osteoclasts and endothelial cells (HMEC-1) and demonstrated that the ceramic material supports cell attachment and differentiation. A significant difference of proliferation of HOB on day 7 compared to a blank control was not established. Additionally, cell attachment and proliferation/differentiation appeared to be higher in comparison to CaSiO_3_ ceramics [21]. By exposing human adipose tissue-derived stem cells (ASCs) and primary osteoblasts (HOBs) to Baghdadite and Hydroxyapatite scaffolds, the group of Lu et al. reported an improved promotion of the osteogenic differentiation in the Baghdadite group [29]. While our study provides results of the effects of a Baghdadite ceramic on the viability and proliferation rate of macrophages, the publication of Graney et al. reports an in vitro modulation of macrophage behavior. Baghdadite appears to affect the M1-to-M2 transition to a similar ratio observed in normal healing and thereby may aid in bone repair [30]. In regard to our results and the available literature, Baghdadite appears to have a sufficient biocompatibility in an in vitro setting to warrant further in vivo investigation.

In this study, we also investigated the proinflammatory potential of Baghdadite on THP-1 and MG-63 cells. MG-63 cells did not exhibit any significant increase or upregulation in the secretion of IL-1ß, IL-6 and TNFα. THP-1-derived macrophages, however, in the presence of Baghdadite, showed an upregulated production of IL-6 and TNFα. Only the upregulation of the production of TNFα proved to be statistically significant. To our best knowledge no other studies investigated the effect of Baghdadite on the secretion of proinflammatory markers such as IL-1ß, IL-6 and TNFα. Therefore, comparability with other results cannot be achieved.

IL-1ß is one of the essential proinflammatory mediators promoting osteolysis in bone–implant interfaces [26]. A dramatic increase in IL-1ß can be achieved through stimulation with LPS, mimicking the situation of an additional low-grade infection and further aggravating inflammation. In an aseptic revision case, a clinical effect may not be as drastic, but needs to be kept in mind. An increase in IL-1ß might also be caused by a simultaneous increase in TNFα, which can act as a priming factor. Such combined increases have been observed in chronic but aseptic low-grade periprosthetic inflammation [31]. TNFα is considered a master cytokine during inflammation and can induce other proinflammatory chemokines and cytokines. According to our results, Baghdadite does possess a proinflammatory capacity due to its induction of TNFα. Whether this is clinically relevant and alters or inhibits bone formation is unclear. The IL-6 cytokine family displays almost contrasting roles and can stimulate the expression of RANKL by osteoblasts to promote the formation of bone-resorbing osteoclasts, but it can also stimulate osteoblast differentiation and promote bone formation [27]. A clinically relevant negative effect on bone formation due to IL-6 induction caused by the presence of Baghdadite needs further investigation.

Baghdadite ceramics present an interesting bone composite material and after evaluating biocompatibility and inflammatory potential in vitro, an in vivo evaluation fitted to the field of RTHA is the logical next step. In vivo, animal models have been published in the literature with promising first results. An interesting publication by the group around Luo evaluated the osteogenic potential of Baghdadite microspheres in a similar setting to impaction bone grafting, as described in the introduction. Ceramic spheres were implanted into the supracondylar region of the femur in Wistar rats. In an immunohistochemical evaluation, Baghdadite presented with enhanced osteogenic potential when compared to silicate-based diopside and ß-tricalcium phosphate [32]. Another investigation was launched by Roohani-Esfahani et al. repairing a critical-sized, segmental bone defect of the radius with a highly porous Baghdadite scaffold in rabbits. Micro-computed tomography and histological analysis after 12 weeks under normal load showed extensive new bone formation with complete bridging of the radial defect [33]. The first large animal model was carried out and published by Li et al. In this study, critical-sized, segmental defects of sheep tibia were treated with Baghdadite scaffolds and analyzed after 26 weeks through electron microscopy, multiphoton microscopy, and histology. The authors report extensive new bone formation that directly abuts the implant surfaces. Furthermore, no evidence of chronic inflammation of fibrous capsule formation, both common with conventional bulk allografts, have been found [34]. A next progression could be the treatment of an uncontained defect and/or a contained defect in a large animal model in combination with a hip revision implant. However, there remains some concern regarding the mechanical strength of Baghdadite as bulk and porous scaffolds. Although the Baghdadite scaffolds have reportedly reached the lower end of the range for cortical and trabecular bone, application for high load-bearing musculoskeletal applications may still be limited [12]. Further biomechanical investigation is required in the future.

## 5. Conclusions

In summary, our investigation of the biocompatibility of Baghdadite ceramics, which was based on testing the viability and proliferation rate of THP-1-derived macrophages and the human osteosarcoma cells MG-63, presented with no significant differences when compared to a control group. We therefore conclude that, in our in vitro setup, no relevant cytotoxicity of Baghdadite ceramics was encountered. However, Baghdadite ceramics exhibit a proinflammatory potential by significantly increasing the secretion of TNFα in THP-1-derived macrophages. Whether this proinflammatory potential results in a clinically relevant inhibition of bone ingrowth is unclear and requires further investigation. Baghdadite ceramics provide an interesting alternative to conventional bone substitutes and should be further investigated. Before a large animal model can be carried out, further biomechanical testing is needed to establish sufficient evidence regarding the required stability for an application in the treatment of bone defects in RTHA.

## Data Availability

Not applicable.
